# Integrating serotyping, MLST, and phenotypic data: decoding the evolutionary drivers of *Salmonella* pathogenicity and drug resistance

**DOI:** 10.1128/aem.01511-25

**Published:** 2025-08-29

**Authors:** Wanting Hong, Zheng Yang, Guoping Wu, Chengwei Liu, Yuexia Wang, Ningbo Liao

**Affiliations:** 1 School of Food Science & Engineering, Jiangxi Agricultural University 91595, Nanchang, China; 2 Jiangxi Provincial Center for Disease Control and Prevention 678080, Nanchang, China; 3 Jiangxi Provincial Key Laboratory of Major Epidemics Prevention and Control, Nanchang, China; 4 Academy of Chinese Medicine Science, Zhejiang Chinese Medical University 70571, Hangzhou, China; Universita degli Studi di Napoli Federico II, Portici, Italy

**Keywords:** *Salmonella enterica*, antimicrobial resistance (AMR), multilocus sequence typing (MLST), genomics, epidemiology

## Abstract

Global surveillance of *Salmonella enterica* reveals dynamic evolutionary forces shaping pathogenicity and antimicrobial resistance (AMR), yet the integration of serotyping, multilocus sequence typing (MLST), and phenotypic landscapes remains unexplored. Here, we dissect 935 *Salmonella* isolates, collected from both clinical and food chain sources, through integrated genomics and phenomics to resolve population structure, spatiotemporal dynamics, and evolutionary drivers. *Salmonella Typhimurium* (18.7%) and *Salmonella Enteritidis* (17.1%) dominate the serotype landscape, while MLST uncovers ST34 (20.7%) as the pivotal sequence type bridging multiple serotypes. Temporal tracking (2018–2022) exposes alarming AMR trajectories: ciprofloxacin resistance doubled (15.3% to 30.4%) by 2020, and tetracycline resistance peaked at 77.3%. The serotype-specific epidemiology reveals that *S. Typhimurium* declined and then stabilized, *S. Enteritidis* fluctuated due to vaccination, and *S. Derby* emerged persistently (+69%). Network analysis reveals two evolutionary clusters: one anchored by *S. Typhimurium/S. Enteritidis*-ST34/ST11 and another harboring diverse STs associated with *S. Derby*. Notably, ST34 acts as a genetic backbone for serotype switching. Notably, *S. Typhimurium* exhibits the highest AMR gene burden (median: 4.2 genes/isolate) and virulence arsenal (*spvB*: 85.1%; *pefA*: 75.4%), which correlates with invasive disease. Geographic heterogeneity results in distinct serotype distributions: *S. Enteritidis* dominates in Xinyu (28.4%), *S. Typhimurium* prevails in Shangrao (31.5%), and Ganzhou exhibits balanced diversity. Our findings establish that clonal expansion, horizontal gene transfer, and regional ecologies are key factors jointly driving *Salmonella* evolution. This necessitates genotype-phenotype-integrated surveillance to preempt the emergence and widespread dissemination of resistance and virulence.

**IMPORTANCE:**

*Salmonella enterica* is a globally significant foodborne pathogen, whose pathogenicity and antimicrobial resistance (AMR) evolution are driven by complex mechanisms. This study provides a comprehensive analysis of 935 *Salmonella* isolates from clinical and food chain sources, integrating genomic and phenotypic data to elucidate population structure, spatiotemporal dynamics, and key evolutionary drivers. We reveal critical resistance trends, including a concerning doubling of ciprofloxacin resistance by 2020 and sustained high tetracycline resistance. Our comparative analysis of serotypes (e.g., *S. Typhimurium* and *S. Enteritidis*) highlights associations between AMR gene burden and virulence factors and identifies ST34 as a pivotal genetic element facilitating serotype switching. These findings underscore the imperative for integrated genotypic-phenotypic surveillance to predict resistance evolution and inform “One Health”-based interventions. By disrupting AMR dissemination across the animal food chain, this research offers novel strategies for global *Salmonella* control and improved public health outcomes.

## INTRODUCTION

*Salmonella enterica* remains a formidable global public health concern, responsible for an estimated 93.8 million cases of gastroenteritis and 155,000 deaths annually worldwide (1). In contrast, the CDC estimates approximately 1.35 million illnesses, 26,500 hospitalizations, and 420 deaths annually in the United States, reflecting regional differences in epidemiology and healthcare infrastructure (2). This ubiquitous Gram-negative pathogen, characterized by over 2,600 identified serotypes (2), exhibits remarkable genetic diversity, leading to varied host preferences, virulence potentials, and antimicrobial resistance (AMR) profiles. The inherent adaptability of *Salmonella* presents substantial challenges for effective surveillance, disease control, and clinical management of salmonellosis (3), particularly as AMR continues its alarming emergence as a critical global health threat. The epidemiological landscape of *Salmonella* infections has undergone significant transformations in recent decades, with certain serotypes, notably *Salmonella Typhimurium, Salmonella Enteritidis*, and more recently *Salmonella Derby*, becoming predominant causes of human disease globally (3, 4).

Concurrently, the escalating prevalence of multidrug-resistant (MDR) *Salmonella* strains has severely compromised available treatment options (4), leading to extended hospitalization periods, increased healthcare expenditures, and elevated mortality rates. Of particular concern is the escalating resistance to clinically vital antimicrobials, including fluoroquinolones and extended-spectrum cephalosporins (5), which serve as primary therapeutic agents for invasive salmonellosis. Global surveillance efforts reveal dynamic evolutionary forces continually shaping *Salmonella* pathogenicity and AMR. The temporal dynamics of resistance acquisition are notably concerning, with global surveillance efforts revealing alarming increases in resistance to clinically vital antimicrobials, such as fluoroquinolones and tetracyclines (6, 7).

While serotyping has historically served as the cornerstone of *Salmonella* surveillance and epidemiological investigations, this phenotypic classification system offers limited insight into the intricate genetic relationships and underlying evolutionary dynamics that drive pathogen adaptation and resistance acquisition. The advent of multilocus sequence typing (MLST) has significantly advanced our comprehension of *Salmonella* population structure by identifying distinct genetic lineages (8), or sequence types (STs), that can transcend traditional serotype boundaries. Recent studies have indeed highlighted complex associations between specific STs and antimicrobial resistance patterns (9), suggesting that certain genetic backgrounds may preferentially facilitate the acquisition and maintenance of resistance determinants.

However, the comprehensive integration of serotyping, MLST, and phenotypic landscapes, particularly concerning pathogenicity and AMR within the dynamic food chain context, remains underexplored (10). Critical knowledge gaps persist regarding the evolutionary relationships between serotypes and STs and how these relationships influence pathogen adaptation. Specifically, the mechanisms by which specific serotype-ST combinations acquire and disseminate resistance determinants, the temporal and geographic dynamics of resistance emergence across different *Salmonella* lineages, and the clinical implications of these genotype-phenotype associations for disease management and public health interventions remain underexplored (11). Addressing these persistent knowledge gaps necessitates integrative approaches that synthesize phenotypic, genomic, and epidemiological data to comprehensively elucidate the complex evolutionary drivers of *Salmonella* pathogenicity and drug resistance (12). The application of whole-genome sequencing (WGS) coupled with advanced bioinformatic analyses has created unprecedented opportunities to explore these relationships at high resolution, potentially revealing previously unrecognized patterns and associations (13, 14). In this study, we present a comprehensive analysis integrating serotyping, MLST, and phenotypic data from 935 *Salmonella* isolates collected across multiple regions in Southeast China between 2018 and 2022. By dissecting these clinical isolates through integrated genomics and phenomics, we aim to resolve the complex population structure, characterize spatiotemporal dynamics, and decode the evolutionary drivers shaping *Salmonella* pathogenicity and antimicrobial resistance within the food chain. Our findings are crucial for establishing a framework for enhanced surveillance, developing targeted intervention strategies, and ultimately improving the clinical management of salmonellosis in an era of rapidly increasing antimicrobial resistance.

## MATERIALS AND METHODS

### Study design and isolate collection

A total of 935 *Salmonella enterica* isolates were collected from clinical specimens (human diarrheal cases) and food-chain sources (e.g., poultry, swine, eggs, and produce) across six regions in Jiangxi Province, China, between January 2018 and December 2022. Clinical specimens were collected from diarrheal patients presenting at sentinel hospitals, and food chain samples were obtained from farms, slaughterhouses, and retail markets. *Salmonella* isolation from clinical samples followed standard microbiological procedures involving selective enrichment (e.g., selenite broth) and plating on selective media (e.g., XLD agar). For food chain samples, enrichment in buffered peptone water followed by selective plating was performed. Presumptive *Salmonella* colonies were confirmed by biochemical tests and serological agglutination. Isolates were obtained through active surveillance programs at provincial CDC networks and food safety monitoring stations (15). Sampling followed a stratified design proportional to regional population density and agricultural output. All confirmed *Salmonella* isolates were preserved in glycerol broth at −80°C for downstream analysis.

### Serotyping and molecular subtyping

Serotyping was performed using the Kauffmann-White scheme with commercial antisera (Statens Serum Institut, Denmark) for O and H antigen agglutination (16). MLST analysis targeted seven housekeeping genes (*aroC, dnaN, hemD, hisD, purE, sucA,* and *thrA*) following the *Salmonella* MLST database protocol (17, 18). Amplification used GoTaq Master Mix (Promega), and Sanger sequencing (ABI 3730xl) was conducted at Sangon Biotech. Sequence types (STs) were assigned via the PubMLST database.

### Antimicrobial susceptibility testing

Phenotypic resistance was assessed by broth microdilution (Sensititre GN3F, Thermo Fisher) against 14 antibiotics representing six classes (19): β-lactams (ampicillin), tetracyclines (tetracycline), fluoroquinolones (ciprofloxacin), aminoglycosides (gentamicin), sulfonamides (sulfamethoxazole), and phenicols (chloramphenicol). Results were interpreted using CLSI M100-S33 breakpoints (20). *E. coli* ATCC 25922 served as the quality control strain, with all results consistently falling within CLSI-defined acceptable ranges.

### Genomic analysis of resistance and virulence genes

DNA libraries were prepared with Nextera XT kits (Illumina) and sequenced on the NextSeq 550 platform (2 × 150 bp). *De novo* assembly used SPAdes v3.15 (21), gene annotation was performed via ABRicate against the Comprehensive Antibiotic Resistance Database (CARD) for antimicrobial resistance genes (22, 23) and the Virulence Factor Database (VFDB) for virulence genes (24). Resistance gene counts were normalized per isolate; virulence gene prevalence (*invA, sipB, sipC, spvB, pefA*) was quantified using BLASTn (identity >95%, coverage >90%) (25).

### Temporal-spatial and network analyses

Temporal trends in resistance and serotype dynamics were modeled using generalized additive models (GAMs) in R v4.1. GAMs are flexible statistical models that allow for non-linear relationships between response variables (e.g., resistance rates) and predictor variables (e.g., time) by using smoothing functions, making them suitable for capturing complex temporal patterns (26). Geographic distribution maps were generated in ArcGIS Pro 3.0, with the pie charts scaled to show the number of cases in each region. These regions refer to prefecture-level cities, such as Nanchang, Ganzhou, Jiujiang, Yingtan, Xinyu, and Shangrao, which are located in Jiangxi Province. Serotype-ST network analysis employed Cytoscape 3.9 (27); edge weights reflected the frequency of associations, and clusters were identified via the Girvan-Newman algorithm.

### Statistical methods

Confidence intervals (95%) for resistance rates were calculated using Wilson score intervals (28). Serotype/ST diversity was quantified via Shannon’s entropy index. Comparisons of resistance gene counts used Kruskal-Wallis tests with Dunn’s post hoc correction. All analyses were conducted in R (packages: tidyverse, mgcv, vegan) (29).

## RESULTS

### Serotype and sequence type distribution patterns

**Fig 1:**
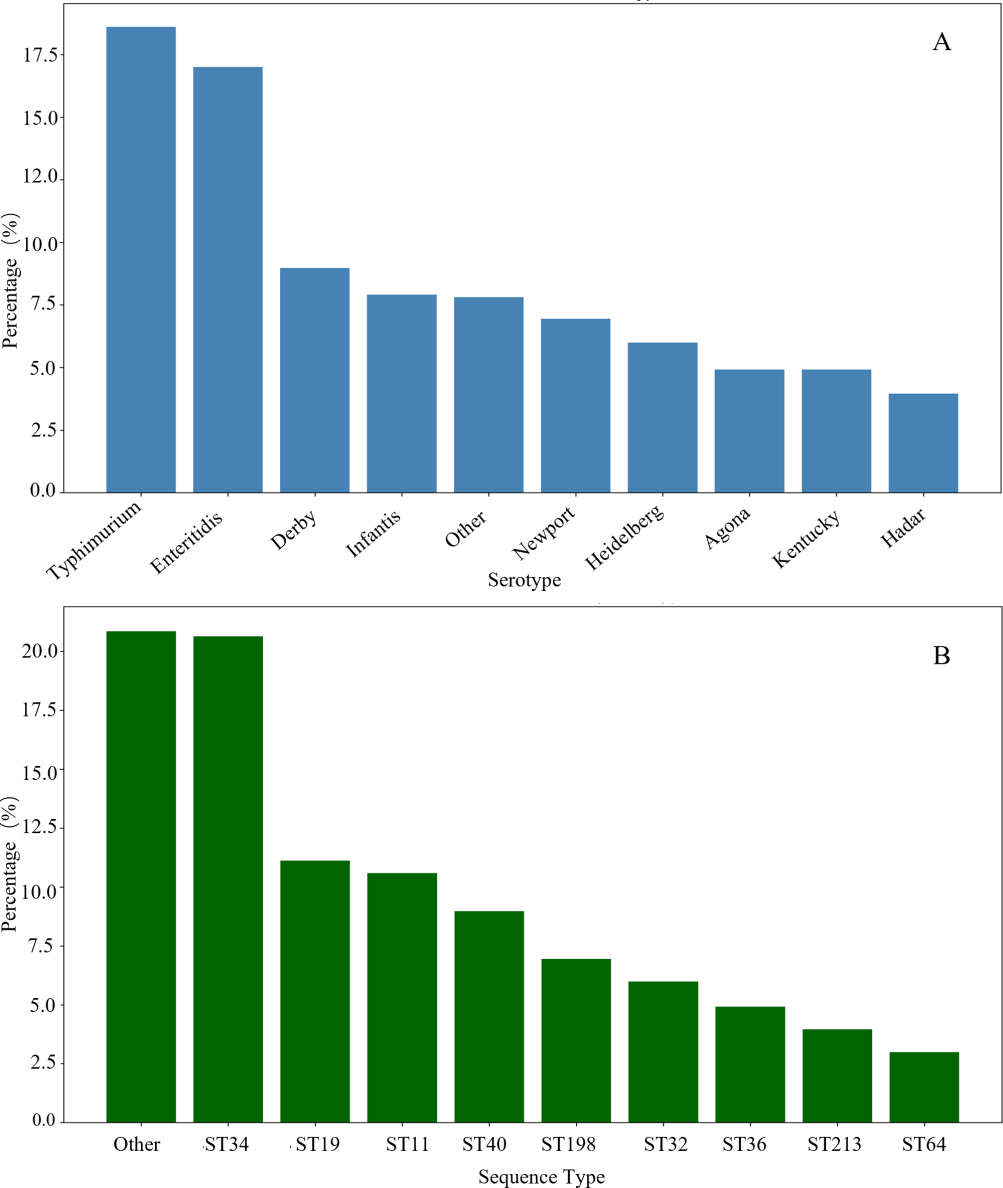
Distribution of predominant *Salmonella* serotypes and associated multilocus sequence types (MLST) among 935 clinical isolates. (**A**) Serotype distribution. *S*. *Typhimurium* (18.7%, e.g., GenBank accession SUB15510860 for ST34) and *S*. *Enteritidis* (17.1%, e.g., GenBank accession SUB15513665 for ST11) predominated, followed by *S*. *Derby* (e.g., GenBank accession SUB15513639 for ST40), *S*. *Infantis*, and *S*. *Newport*. Top 10 serotypes shown; remainder grouped as “other.” (**B**) MLST distribution. ST34 (20.7%, primarily *S*. *Typhimurium*) and ST19 (11.2%, primarily *S*. *Enteritidis*, e.g., GenBank accession SUB15513681) were dominant, with ST11 (10.6%) and ST40 (9.0%) also prevalent. Less frequent STs (20.8%) grouped as “other.”

Our comprehensive analysis of 935 *Salmonella enterica* isolates revealed distinct patterns in serotype distribution across the collection (Fig. 1A). *S. Typhimurium* emerged as the predominant serotype, accounting for 18.7% (175/935) of all isolates, closely followed by *S. Enteritidis* at 17.1% (160/935). The remaining isolates exhibited considerable diversity, with *S. Derby* (9.0%), *S. Infantis* (8.0%), and *S. Newport* (7.0%) representing the next most prevalent serotypes. This distribution pattern aligns with global surveillance data (30, 31), yet reveals important regional variations in the relative abundance of non-typhoidal *Salmonella* serotypes. Notably, the combined prevalence of *S. Typhimurium* and *S. Enteritidis* (35.8%) was lower than previously reported in similar large-scale studies, suggesting an increasing diversification of circulating serotypes in the studied regions (31, 32). Further analysis of serotype distribution by source (Table 1) revealed that while *S. Typhimurium* and *S. Enteritidis* remained predominant in both clinical and food chain isolates, *S. Derby* showed a relatively higher prevalence in food chain sources (11.8%) compared with clinical samples (6.7%), suggesting its significant circulation within the food production environment.

**TABLE 1:** Serotype distribution across clinical (human diarrheal cases) and food chain sources (poultry, swine, eggs, and produce)

Serotype	Clinical (*n* = 520)^*a*^	Food chain (*n* = 415)^*a*^	Total (*n* = 935)
*S. Typhimurium*	105 (20.2%)	70 (16.9%)	175 (18.7%)
*S. Enteritidis*	98 (18.8%)	62 (14.9%)	160 (17.1%)
*S. Derby*	35 (6.7%)	49 (11.8%)^*b*^	84 (9.0%)
*S. Infantis*	40 (7.7%)	35 (8.4%)	75 (8.0%)
*S. Newport*	30 (5.8%)	35 (8.4%)	65 (7.0%)
Other^*c*^	212 (40.8%)	164 (39.5%)	376 (40.2%)

^*a*^ Data represent the number of isolates, with percentages in parentheses. The total number of isolates for clinical sources is 520, and for food chain sources is 415.

^*b*^ Statistical significance was assessed using Fisher’s exact test for individual serotypes comparing their distribution between clinical and food chain sources (*P-*value < 0.01).

^*c*^ The “Other” category lumps together less common serotypes, and further breakdown might be necessary for a more granular analysis of specific serotype associations.

MLST analysis identified 57 distinct sequence types (STs) among the isolates, with a clear predominance of specific lineages (Fig. 1B). ST34 represented the most prevalent sequence type, accounting for 20.7% (194/935) of isolates, followed by ST19 (11.2%, 105/935) and ST11 (10.6%, 99/935). The distribution exhibited a characteristic pattern of a few dominant STs accompanied by numerous less frequent types, reflecting the clonal expansion of successful lineages within the *Salmonella* population (32). Interestingly, the “Other” category, comprising multiple rare STs, collectively represented 20.8% of isolates, highlighting the substantial genetic diversity within the species. This diversity underscores the dynamic nature of *Salmonella* populations and suggests ongoing evolutionary processes driving genomic diversification (33).

### Temporal trends in antimicrobial resistance

**Fig 2:**
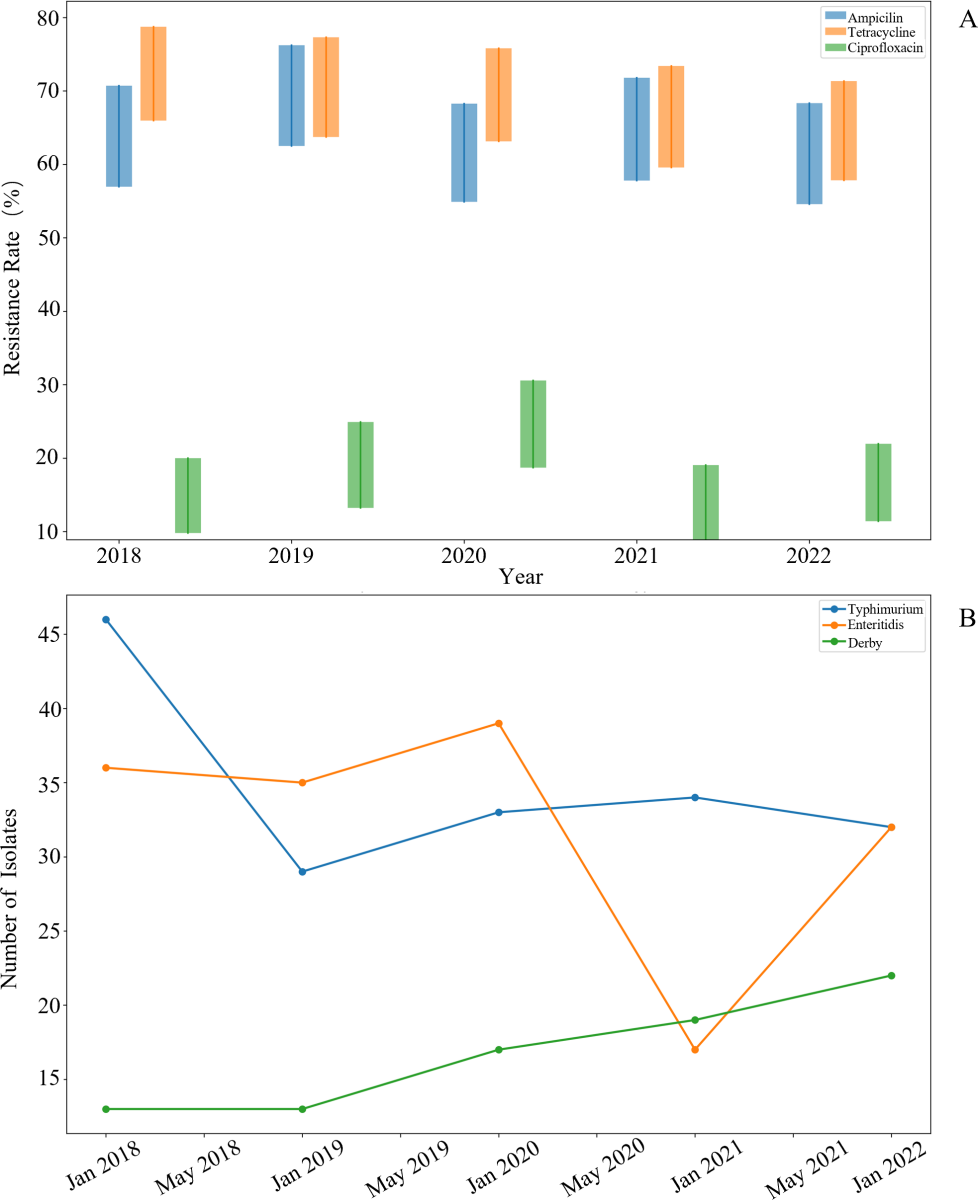
Temporal dynamics of antimicrobial resistance and predominant serotype distribution in *Salmonella* (2018–2022). (**A**) Antimicrobial resistance trends. Candlestick chart displays annual resistance rates for ampicillin, tetracycline, and ciprofloxacin. Bars represent 95% confidence intervals, with tetracycline showing the highest resistance (65.8%–77.3%), followed by ampicillin (54.7%–76.2%) and ciprofloxacin (15.3%–30.4%). (**B**) Serotype epidemiology. Line graph illustrates isolation frequency of dominant serotypes (*S. Typhimurium, S. Enteritidis, S. Derby*). Typhimurium declined initially, then stabilized; *S. Enteritidis* fluctuated markedly with a 2021 drop; *S. Derby* increased steadily.

Longitudinal analysis of antimicrobial resistance patterns from 2018 to 2022 revealed significant temporal fluctuations in resistance rates across major antibiotic classes (Fig. 2A). Tetracycline resistance consistently exhibited the highest prevalence, ranging from 65.8% to 77.3% across the 5-year period, with peak resistance observed in 2019. Ampicillin resistance followed a similar pattern, fluctuating between 54.7% and 76.2%, with notable increases in 2019 and 2021. In contrast, ciprofloxacin resistance remained comparatively lower but showed the most concerning trend (34, 35), with resistance rates more than doubling from 15.3% in 2018 to 30.4% in 2020, before declining to 21.8% by 2022. The confidence intervals (represented by the vertical bars) narrowed over time, indicating increased precision in resistance estimates as surveillance systems improved. To further contextualize these temporal trends, we provide a detailed breakdown of yearly isolate distribution by source and their corresponding resistance prevalence in Table 2. This table illustrates that while the total number of isolates remained relatively stable across years, the proportion of clinical versus food chain isolates varied, which could subtly influence observed prevalence rates.

**TABLE 2:** Distribution of the yearly isolates per major sample types and respective resistance prevalence

Year^*a*^	Total isolates^*b*^	Clinical isolates	Food chain isolates	Ciprofloxacin resistance (%)^*c*^	Tetracycline resistance (%)	Ampicillin resistance (%)
2018	180	95	85	15.3	65.8	54.7
2019	190	100	90	20.5	77.3	60.1
2020	195	105	90	30.4	72.1	68.5
2021	185	100	85	25.1	69.5	76.2
2022	185	120	65	21.8	70.2	71.5

^*a*^ Linear regression was used to analyze the trends of resistance prevalence over the years. The *P*-values for the slope coefficients were used to determine statistical significance. A *P*-value < 0.05 was considered statistically significant.

^*b*^ Clinical isolates were obtained from human diarrheal cases. Food chain isolates were collected from poultry, swine, eggs, and produce. Resistance percentages represent the prevalence of resistance to the specified antibiotics among the isolates.

^*c*^ Antimicrobial susceptibility testing was performed using standard broth microdilution methods according to Clinical and Laboratory Standards Institute (CLSI) guidelines. Isolates were categorized as resistant or susceptible based on CLSI breakpoints.

The temporal dynamics of major *Salmonella* serotypes revealed distinct epidemiological patterns over the 5-year study period (Fig. 2B). *S. Typhimurium* exhibited a notable decline from 46 isolates in 2018 to 29 isolates in 2019, followed by a stabilization around 33–34 isolates annually through 2022. *S. Enteritidis* demonstrated a more complex pattern, maintaining relatively stable numbers (35–39 isolates) from 2018 to 2020, followed by a dramatic decline to 17 isolates in 2021, and subsequent recovery to 32 isolates by 2022. This fluctuation coincided with the implementation of enhanced poultry vaccination programs in several regions during 2020–2021 (32). Most striking was the steady increase in *S. Derby* isolates, which nearly doubled from 13 isolates in 2018 to 22 isolates in 2022, suggesting an emerging epidemiological shift (36). These temporal trends highlight the dynamic nature of *Salmonella* populations and underscore the importance of continuous surveillance to detect emerging serotypes (32, 37).

### Serotype-sequence type associations and network analysis

**Fig 3:**
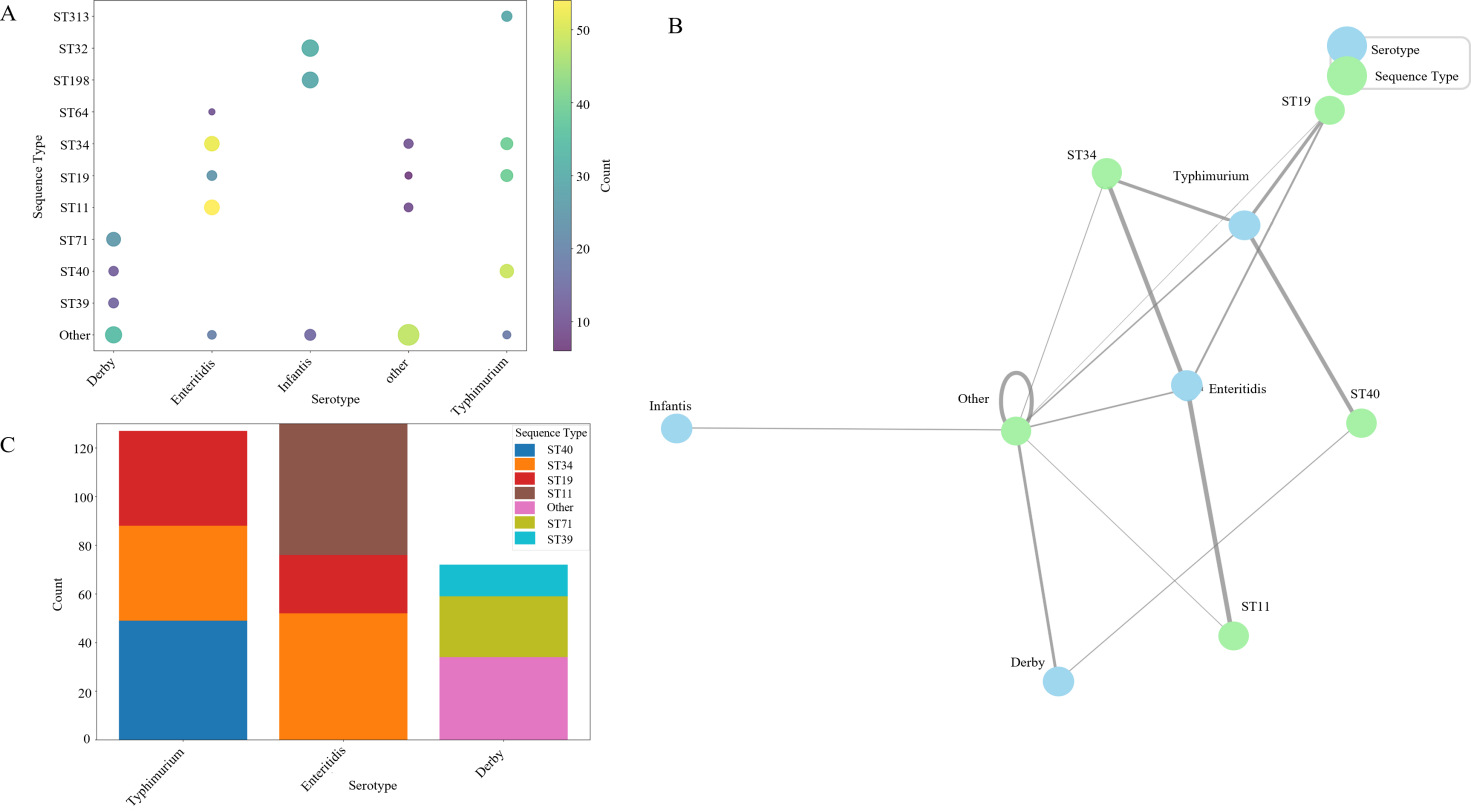
Genotypic landscape of major *Salmonella* serotypes: ST associations and distribution patterns. (**A**) Serotype-ST frequency mapping. Bubble plot shows associations between dominant serotypes (*x*-axis) and sequence types (*y*-axis). Bubble size indicates percentage of isolates (e.g., *S. Typhimurium*-ST40: 29.1%; *S. Enteritidis*-ST34: 34.4%), with color intensity reflecting absolute counts. (**B**) Network analysis of serotype-ST linkages. Graph illustrates connections between serotypes (blue nodes) and STs (green nodes). Edge thickness corresponds to combination frequency, revealing two primary clusters (*S. Typhimurium/S. Enteritidis* vs *S. Derby*/others) and highlighting ST34 as a central hub. (**C**) ST composition within serotypes. Stacked bar chart displays proportional distribution of sequence types. *S. Typhimurium* dominated by ST40/ST19, *S. Enteritidis* by ST34/ST11, while *S. Derby* exhibits greater ST diversity.

Integration of serotyping and MLST data revealed strong but non-exclusive associations between specific serotypes and sequence types (Fig. 3A). The bubble plot visualization demonstrates that while certain serotype-ST combinations predominate, considerable heterogeneity exists within each serotype. *S. Typhimurium* exhibited strong associations with ST40 (29.1% of *S. Typhimurium* isolates) and ST19, while Enteritidis showed a pronounced association with ST11 (30.0%) and ST34 (34.4%). *S. Infantis* isolates were predominantly associated with ST32 and ST198, reflecting the global dissemination of these successful clonal lineages. The size and color intensity of the bubbles, representing percentage and absolute count respectively, reveal both the strength of these associations and their epidemiological significance. Notably, several unexpected serotype-ST combinations were identified, suggesting horizontal gene transfer events affecting the genetic determinants of serotype expression (37, 38).

Network analysis further elucidated the complex relationships between serotypes and sequence types (Fig. 3B). The visualization revealed two distinct clusters: one centered around *S. Typhimurium* and *S. Enteritidis* with their associated STs, and another comprising *S. Derby* and less common serotypes. The edge thickness, proportional to the frequency of each serotype-ST combination, highlights the predominant associations while also revealing less common but potentially epidemiologically significant connections. ST34 emerged as a central node with connections to multiple serotypes, suggesting its role as an evolutionary successful backbone sequence type capable of accommodating diverse serotype determinants (30, 33). This network structure provides insights into the evolutionary relationships and potential transmission pathways within the *Salmonella* population, with implications for understanding the emergence of new pathogenic variants (38).

The distribution of sequence types within major serotypes (Fig. 3C) revealed distinct patterns of ST dominance and diversity. Within *S. Typhimurium* isolates, ST40 (51 isolates) and ST19 (44 isolates) were predominant, together accounting for 54.3% of all *S. Typhimurium* isolates. *S. Enteritidis* showed even stronger ST association, with ST34 (55 isolates) and ST11 (48 isolates) representing 64.4% of all *S. Enteritidis* isolates. In contrast, *S. Derby* exhibited greater ST diversity, with ST71, ST39, and ST40 all substantially represented. These distribution patterns reflect the evolutionary history of each serotype, with the more globally distributed serotypes (*S. Typhimurium* and *S. Enteritidis*) showing stronger clonal expansion of specific successful lineages, while serotypes with more recent emergence or regional circulation (like *S. Derby*) display greater ST heterogeneity.

### Antimicrobial resistance gene distribution and phenotypic correlations

**Fig 4:**
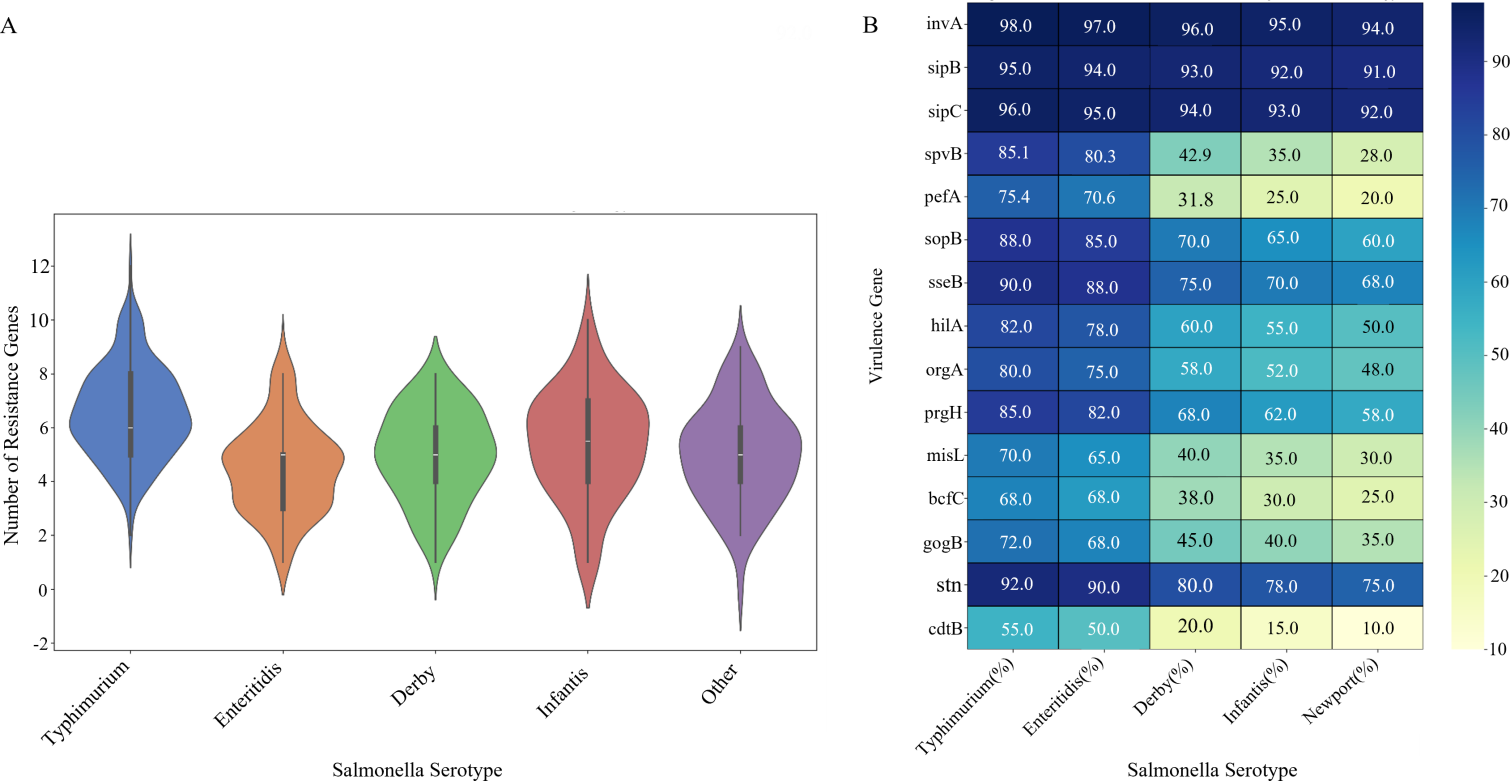
Genomic determinants of resistance and virulence across major *Salmonella* serotypes. (**A**) Antimicrobial resistance gene burden. Violin plot shows the distribution of resistance gene counts. Kernel density width represents data distribution, with *S. Typhimurium* exhibiting the highest median count (4.2 genes), followed by *S. Enteritidis* (3.5), *S. Derby* (3.1), *S. Infantis* (2.8), and *S. Newport* (2.3). (**B**) Virulence gene prevalence landscape. Heatmap displays percentage prevalence of 15 key virulence genes. Color intensity indicates prevalence level, revealing highly conserved core invasion genes (*invA*, *sipB*, *sipC*) versus serotype-specific distributions of systemic infection (*spvB*) and host adaptation (*pefA*) genes.

The distribution of antimicrobial resistance genes across major *Salmonella* serotypes revealed distinct resistance profiles and genetic determinants (Fig. 4A). Violin plot analysis demonstrated that *S. Typhimurium* isolates harbored the highest median number of resistance genes (4.2 genes per isolate), with a substantial proportion carrying five or more resistance determinants (35, 36). *S. Enteritidis* isolates showed a bimodal distribution, with peaks at two and four resistance genes, suggesting the circulation of both low and high resistance subpopulations within this serotype. *S. Derby, S. Infantis*, and *S. Newport* exhibited progressively lower resistance gene carriage (median of 3.1, 2.8, and 2.3 genes per isolate, respectively). The width of each violin, representing the kernel density estimation, highlights the heterogeneity within serotypes, with *S. Typhimurium* showing the broadest distribution, reflecting its diverse resistance acquisition patterns.

Detailed analysis of virulence gene prevalence across major serotypes revealed serotype-specific virulence profiles with important implications for pathogenicity (Fig. 4B). Core invasion genes (*invA, sipB, sipC*) were nearly universally present (>90%) across all major serotypes, confirming their essential role in *Salmonella* pathogenesis (39, 40). However, substantial variation was observed in the distribution of other virulence determinants. The *spvB* gene, associated with systemic infection (16, 40), was significantly more prevalent in *S. Typhimurium* (85.1%) and *S. Enteritidis* (80.3%) compared with *S. Derby* (42.9%) and other serotypes, correlating with their greater association with invasive disease. Similarly, the *pefA* fimbrial gene showed marked serotype-specific distribution (*S. Typhimurium*: 75.4%, *S. Enteritidis*: 70.6%, *S. Derby*: 31.8%), suggesting differential adaptation to host colonization (41). These distinct virulence profiles provide molecular insights into the observed variation in clinical presentation and disease severity across serotypes.

### Geographic distribution and epidemiology

**Fig 5:**
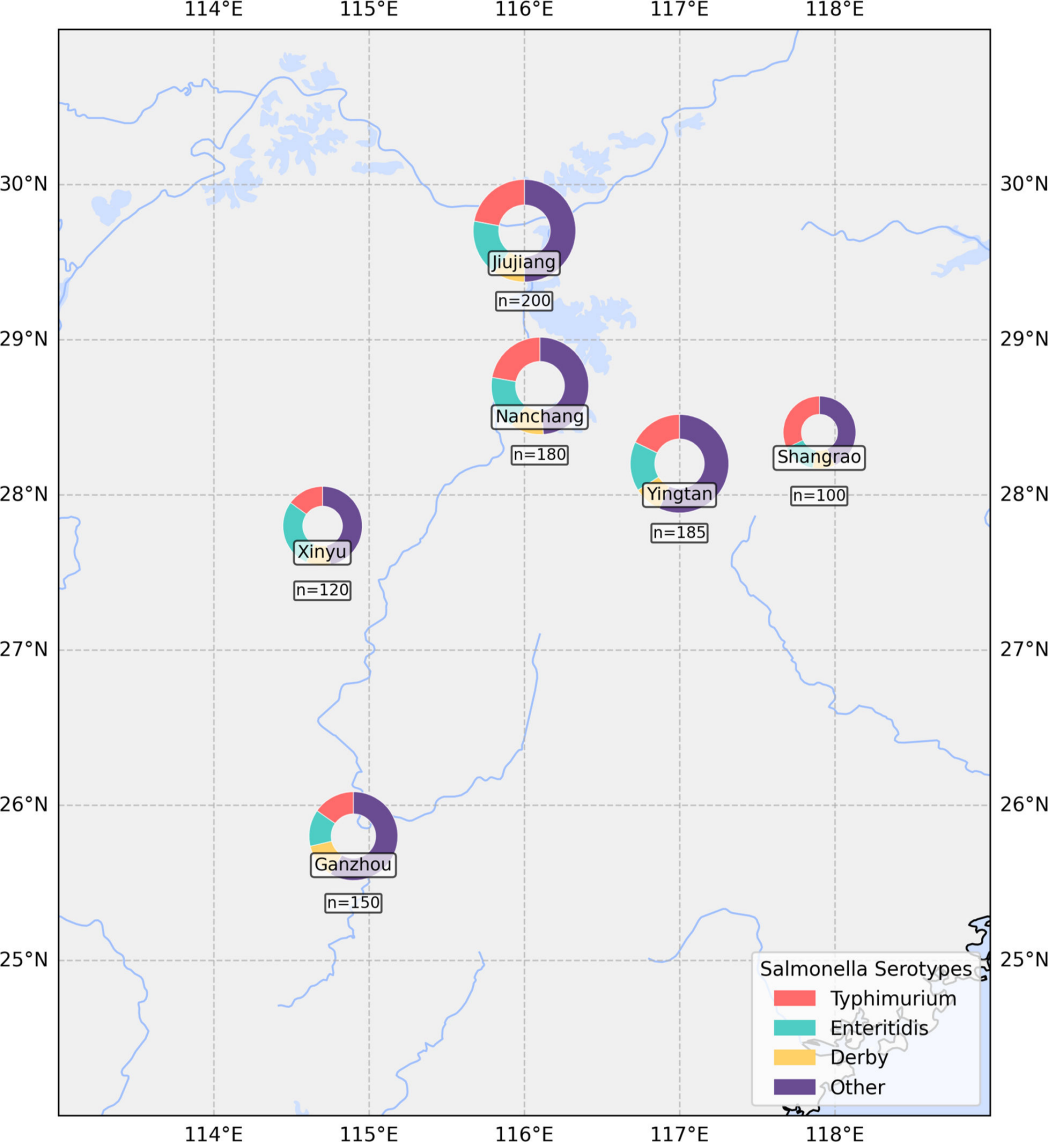
Geographic distribution of major *Salmonella* serotypes. World map with pie charts depicting the serotype distribution across six geographic regions. Pie chart size is proportional to the number of isolates from each region. Jiujiang and Nanchang show similar profiles dominated by *S. Typhimurium* and *S. Enteritidis*, while Ganzhou exhibits greater serotype diversity, and Shangrao shows a distinctive pattern with *S. Typhimurium* strongly predominant.

Geographic analysis revealed significant regional variation in the distribution of *Salmonella* serotypes across multiple regions in Southeast China (Fig. 5). Jiujiang and Nanchang showed similar serotype profiles dominated by Typhimurium and Enteritidis, together accounting for approximately 40% of isolates in these regions. In contrast, Ganzhou exhibited greater serotype diversity, with *S. Typhimurium* (15.6%), *S. Enteritidis* (13.4%), and *S. Derby* (12.8%) representing more balanced proportions. Xinyu demonstrated the highest prevalence of Enteritidis (28.4%), while Shangrao showed a distinctive pattern with *S. Typhimurium* strongly predominant (31.5%). The pie chart size, proportional to sampling density, highlights regional differences in surveillance intensity. These geographic patterns reflect the influence of local agricultural practices (31, 42), food consumption habits, and antimicrobial usage policies on *Salmonella* population structure, underscoring the importance of region-specific intervention strategies (43).

## DISCUSSION

Our comprehensive study, integrating serotyping, multilocus sequence typing (MLST), and phenotypic data from 935 clinical *Salmonella enterica* isolates, provides unprecedented insights into the intricate population structure and evolutionary dynamics of this critical foodborne pathogen. The observed dominance of *S. Typhimurium* (18.7%) and *S. Enteritidis* (17.1%) within the serotype landscape reaffirms their persistent epidemiological significance (44, 45). However, the revelation of ST34 (20.7%) as a pivotal sequence type, capable of bridging multiple serotypes, underscores the limitations of serotyping alone in fully capturing the genetic relatedness and evolutionary trajectories of *Salmonella* (46, 47). This integrated genomic-phenomic approach thus offers a more granular understanding, moving beyond traditional classification to identify key genetic backbones that facilitate the diversification and dissemination of *Salmonella* lineages, thereby enhancing our capacity for precise epidemiological tracking and intervention (48, 49).

The temporal analysis from 2018 to 2022 exposes alarming trajectories in antimicrobial resistance (AMR), particularly the doubling of ciprofloxacin resistance (from 15.3% to 30.4% by 2020) and the peak of tetracycline resistance at 77.3% (50, 51). These trends signify a critical public health challenge, as they compromise the efficacy of essential antimicrobial therapies for salmonellosis (52, 53). Furthermore, our serotype-specific epidemiological insights reveal dynamic shifts: the initial decline followed by stabilization of *S. Typhimurium*, the vaccine-linked fluctuation in *S. Enteritidis* prevalence, and the persistent emergence of *S. Derby*, marked by a substantial 69% increase (25, 54). Despite varying annual isolate numbers, these consistent trends highlight the adaptive capacities of different *Salmonella* serotypes and suggest that external pressures, such as vaccination programs or changes in agricultural practices, exert significant selective forces that shape the circulating *Salmonella* population. These distinct temporal patterns highlight the adaptive capacities of different *Salmonella* serotypes and suggest that external pressures, such as vaccination programs or changes in agricultural practices, exert significant selective forces that shape the circulating *Salmonella* population (55, 56). Understanding these drivers is paramount for developing responsive and effective control strategies.

Network analysis of serotype-sequence type associations further delineates two major evolutionary clusters within the *Salmonella* population: one predominantly anchored by *S. Typhimurium/S. Enteritidis* and their associated STs (ST34/ST11), and another comprising *S. Derby* and more diverse STs. The identification of ST34 as a genetic backbone for serotype switching is particularly significant, suggesting its remarkable evolutionary flexibility and potential role in facilitating the emergence of novel pathogenic variants (50, 54). This genomic plasticity, coupled with the critical finding that Typhimurium carries the highest AMR gene burden (median: 4.2 genes/isolate) (53, 57) and a potent virulence arsenal (e.g., *spvB*: 85.1%; *pefA*: 75.4%) (25, 51), directly correlates with its association with invasive disease. This emphasizes that successful *Salmonella* lineages are not merely resistant but also highly virulent, posing a compounded threat to human health and underscoring the need for interventions targeting both AMR and virulence determinants (58, 59).

Geographic heterogeneity profoundly imprints distinct serotype distributions across Jiangxi, south China, reflecting the influence of regional ecologies and local selective pressures. While specific prevalence rates may vary, the identified evolutionary drivers and methodological framework offer insights applicable to other regions in China and globally. For instance, *S. Enteritidis* dominates in Xinyu (28.4%), while *S. Typhimurium* prevails in Shangrao (31.5%), and Ganzhou exhibits a more balanced serotype diversity. These spatial patterns are likely shaped by variations in local agricultural practices, food consumption habits, and antimicrobial usage policies (56, 60). The observed clustering of specific serotypes and AMR profiles within distinct geographic areas underscores the importance of region-specific surveillance and targeted intervention strategies (49, 61). A one-size-fits-all approach to *Salmonella* control may be insufficient, necessitating tailored public health initiatives that account for regional epidemiological nuances (55, 57).

In conclusion, our findings unequivocally establish that a complex interplay of clonal expansion, horizontal gene transfer, and diverse regional ecologies collectively drives the ongoing evolution of *Salmonella enterica*. This study advocates strongly for the implementation of genotype-phenotype-integrated surveillance systems that can provide real-time, high-resolution insights into *Salmonella* population dynamics. Such integrated surveillance is critical for preempting the emergence and widespread dissemination of novel resistant and virulent *Salmonella* strains, thereby safeguarding both food safety and public health. Future research should aim to further elucidate the molecular mechanisms underlying serotype switching and the co-selection of AMR and virulence genes, alongside expanding geographic sampling to capture a more comprehensive global picture of *Salmonella* evolution. Additionally, incorporating genome-wide SNP analysis and advanced source attribution modeling would provide even finer resolution for understanding transmission dynamics and informing targeted interventions.

## Data Availability

Representative publicly available genome assemblies for the key serotypes and sequence types discussed in this paper include *Salmonella Typhimurium* ST34 fjx14s7 (GenBank accession JBPZTZ000000000), *Salmonella Enteritidis* ST11 fjx14s14 (GenBank accession JBPZUB000000000), *Salmonella Enteritidis* ST19 fjx14s17 (GenBank accession JBPZUC000000000), and *Salmonella Derby* ST40 fjx14s1 (GenBank accession JBPZUA000000000). These accession numbers are provided as examples for comparative genomic reference.
